# Tris DBA palladium is an orally available inhibitor of GNAQ mutant uveal melanoma *in vivo*

**DOI:** 10.18632/oncotarget.27040

**Published:** 2019-07-09

**Authors:** Elgilda Musi, Gary K. Schwartz, Jae Hyuk Yoo, Shannon J. Odelberg, Dean Y. Li, Michael Y. Bonner, Ponniah Selvakumar, Shikha Rao, Linda C. Gilbert, Justin Elsey, Jack L. Arbiser

**Affiliations:** ^1^ Department of Medicine, Columbia University Medical Center, New York, New York, USA; ^2^ Herbert Irving Comprehensive Cancer Center, Columbia University College of Medicine, New York, New York, USA; ^3^ Department of Medicine, Program in Molecular Medicine, University of Utah, Salt Lake City, Utah, USA; ^4^ Department of Neurobiology and Anatomy, University of Utah, Salt Lake City, Utah, USA; ^5^ Department of Human Genetics, University of Utah, Salt Lake City, Utah, USA; ^6^ Department of Internal Medicine, Division of Cardiovascular Medicine, University of Utah, Salt Lake City, Utah, USA; ^7^ Department of Oncological Sciences, University of Utah, Salt Lake City, Utah, USA; ^8^ Department of Dermatology, Emory University School of Medicine, Atlanta, Georgia, USA; ^9^ Department of Pathology and Laboratory Medicine, College of Medicine, University of Saskatchewan, Saskatoon, Saskatchewan, Canada; ^10^ Veterans Affairs Medical Center, Decatur, Georgia, USA

**Keywords:** melanoma, chemotherapy

## Abstract

Uveal melanoma is a rare but often lethal malignancy and is the leading cause of death due to an ophthalmic condition. Uveal melanoma is often diagnosed at a late stage and has a strong propensity to hepatic metastasis. Recently, the most common driver mutations in uveal melanoma have been identified, predominantly in the G-proteins GNAQ. This pattern differs from that of cutaneous melanoma in which Braf and Nras predominate. There are no current clinically used agents that target GNAQ mutations, unlike the use of Braf inhibitors in cutaneous melanoma. We tested the novel agent Tris DBA palladium and found that it was markedly more effective against GNAQ mutant melanomas than wild type uveal melanomas. Given that ARF6 has recently been discovered as a node in GNAQ mutations, we evaluated the efficacy of Tris DBA palladium on ARF6 signaling and found that it was effective in inhibiting ARF6 activation. Finally, Tris DBA palladium was orally effective against GNAQ mutant melanoma *in vivo*. Tris DBA Palladium deserves further evaluation as a systemic agent for uveal melanoma.

## INTRODUCTION

Uveal melanoma is an uncommon malignancy that arises from uveal melanocytes. It differs from cutaneous melanoma in several important ways. First, because of its location, detection is often delayed until the lesion reaches an advanced state, and biopsy is more difficult. Second, it appears to be genetically distinct from cutaneous melanoma, with driver mutations in GNAQ and GNA11 being most common in uveal melanoma, while Braf and Nras are the most common driver mutations in cutaneous melanoma [1, 2]. Finally, uveal melanoma has a distinct propensity to liver metastases, compared with cutaneous metastases, which more commonly metastasize to lymph nodes, lung, and brain [3]. While targeting MAP kinase signaling in cutaneous melanoma with Braf and MEK inhibitors have led to increased survival in cutaneous melanoma [4, 5], similar targeting of signaling pathways lags behind in uveal melanoma.

We have previously demonstrated activity of a novel small molecule, Tris DBA palladium, in preclinical models of cutaneous melanoma and pancreatic cancer. In cutaneous melanoma, we found that Tris DBA palladium inhibits N-myristoyltransferase 1 (NMT1) and blocks tumor growth *in vivo* [6]. In pancreatic carcinoma, we demonstrated that Tris DBA palladium inhibits motility and metastases of orthotopic pancreatic carcinoma to the liver [7]. In this report, we demonstrate that Tris DBA palladium is effective *in vitro* against a panel of human uveal melanoma cell lines with mutations in GNAQ and GNA11. Uveal melanoma without G protein mutations appears less sensitive than GNAQ and GNA11 mutant cells. Surprisingly, we did not observe inhibition of NMT1 protein or activity in treated uveal melanoma cells. Thus, we examined alternative mechanisms of activity of Tris DBA palladium. Recently, ARF6, a small GTPase, has been found to be a major node in GNAQ mutant uveal melanoma [8]. We found that Tris DBA palladium inhibits ARF6 activation in a dose dependent manner in GNAQ mutant melanoma cells. Finally, we discovered that Tris DBA is orally active against GNAQ mutant melanoma *in vivo*. Thus, Tris DBA palladium deserves further evaluation as a targeted therapy in uveal melanoma.

## RESULTS

### Tris DBA palladium inhibits uveal melanoma cell growth

**Figure 1:**
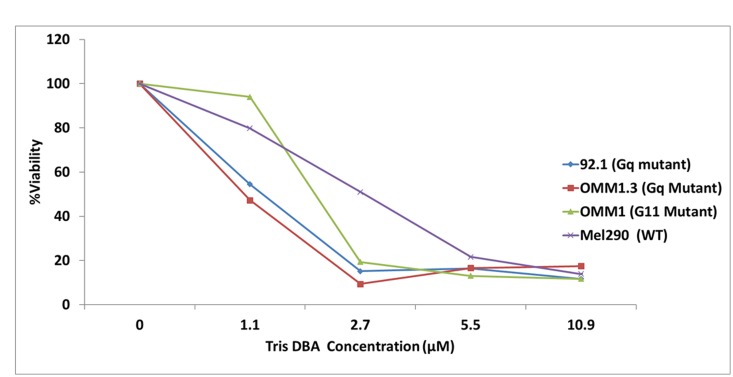
Tris DBA reduces cell viability in GNAQ/GNA11 mutant cell lines. Tris DBA selectively inhibits cell proliferation of GNAQ and GNA11-mutant cells. 92.1(Gq mutant), Omm1.3 (Gq mutant), OMM1 (G11 mutant) and Mel290 (Wild Type) uveal melanoma cell lines were treated with 0, 1.1, 2.7, 5.5 and 10.9 μM of Tris DBA for 24 hours. CCK-8 reagent from Dojindo Molecular Technologies (Rockville, MD, USA), was added after drug treatment to the cells and OD was read at 450 nm. Drug treated samples were presented as percent of control. Results represent the mean of three independent experiments.

A panel of human uveal melanoma cell lines, including mutants in GNAQ, GNA11, and wild type were treated with a dose range of Tris DBA palladium, and cell number was evaluated at 24 hours. All cell lines demonstrated sensitivity to Tris DBA palladium, but the GNAQ and GNA11 cell lines were relatively more sensitive than the wild type melanoma cells (Mel290) to the drug. The GI50 value was 1.1 μM for the GNAQ and GNA11 mutant cell lines and 2.7 μM for the wild type cell line (Figure 1).

### Tris DBA palladium inhibits tumor growth in GNAQ xenograft model

**Figure 2:**
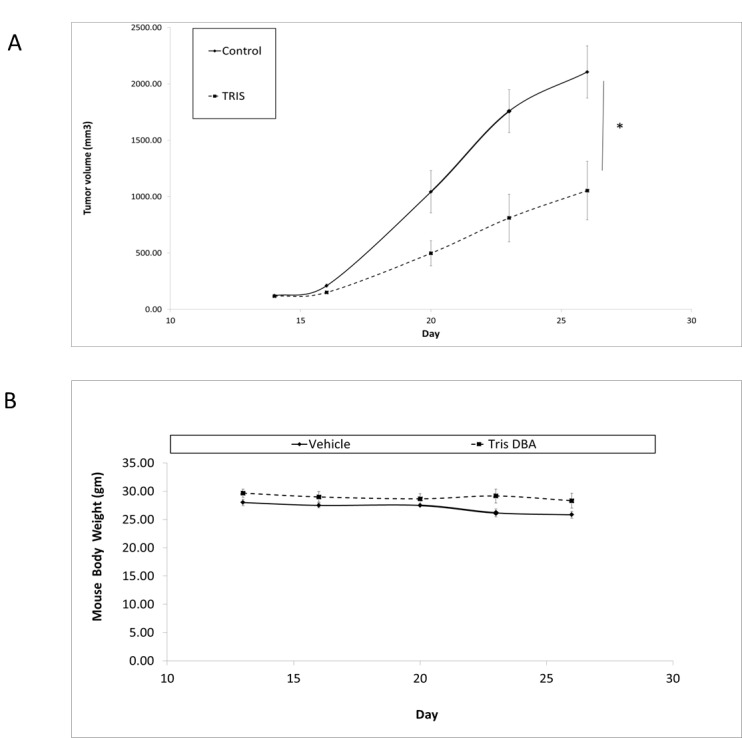
Tris DBA inhibits *in vivo* tumor growth in a GNAQ mutant xenograft model. (**A**) Tris DBA inhibited tumor growth in a uveal GNAQ xenograft model. 6–8 week nu/nu SCID female mice were subcutaneously injected with 92.1 uveal melanoma cells. Tris DBA feed began after tumors reached 100 mm^3^ for a total of two weeks. Tumors were measured with calipers every 2 to 3 days. Tumor volume was compared between groups of mice at various points in time. ^*^ *P*-value when compared to Tris DBA was *p* = 0.01 at day 26. (**B**) Mice body weights were used as measurement of toxicity.

In view of Tris DBA effects observed *in vitro*, we elected to determine whether this therapy is effective in a GNAQ mutant xenograft mouse model. As shown in Figure 2A, Tris DBA had significant effect on inhibiting tumor growth with more than 50% inhibition. The therapy resulted in a significantly enhanced reduction in tumor volume (*p* = 0.01 at day 26) when compared to vehicle control. Toxicity was measured along with tumor volume by weight loss, which was less than 10% for all treatments (Figure 2B).

### N-myristoyltransferase activity is not inhibited in uveal melanoma cells

**Figure 3:**
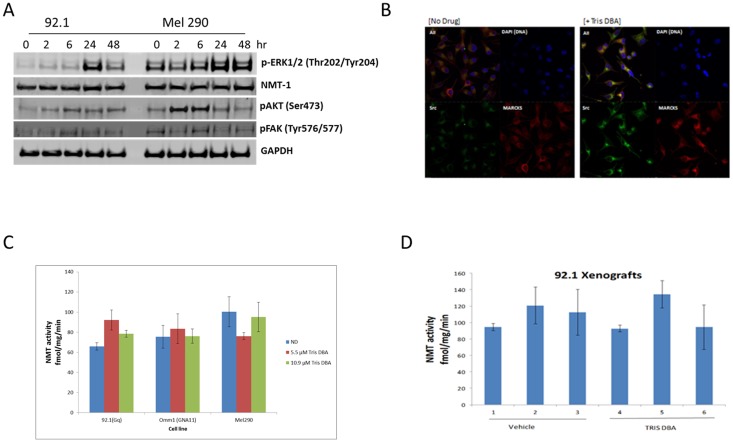
Tris DBA inhibits uveal melanoma tumor growth independent of NMT1. (**A**) Tris DBA does not inhibit MAPK, AKT or FAK pathways in GNAQ uveal melanoma cells. Western Blot of phospoho-ERK1/2 (Thr202/Tyr204), NMT-1, phosphor-AKT (Ser473) and phospho-FAK (Tyr397) at 0, 2, 6, 24 and 48 hours is shown at 2.7 μM Tris DBA. GAPDH was used as a loading control. Briefly, 92.1(Gq mutant) and Mel290 (Wild Type) uveal melanoma cell lines were treated with Tris DBA and lysates were collected in RIPA buffer. Protein concentrations were determined and 30μg of protein was loaded onto a gradient gel. Western Blot was then performed on proteins of interest. (**B**) Immunofluorescence of 92.1 uveal melanoma cells showing expression of SRC and MARCKS following drug treatment at 2.7 μM for 24 hours. Briefly, cells were treated with Tris DBA for 24 hours, after fixation cells were then incubated with primary antibodies overnight at 4° C. Next day, cells were incubated in fluorescently conjugated secondary antibody and mounted onto slides. (**C**, **D**) NMT1 activity was assayed in uveal melanoma cell lines. 92.1(Gq mutant), OMM1 (G11 mutant) and Mel290 (Wild Type) and 92.1 xenografts presenting no inhibition of NMT-1 with drug treatment. Briefly, 20 μg of total protein lysate was used and the myristolation reaction was initiated by the addition of freshly generated [3H]myristoyl-CoA. The samples were incubated 30° C for 30 min. The reaction was terminated and radioactive incorporation was determined by the liquid scintillation counting.

As previously reported, Tris DBA has been shown to inhibit MAPK, PKC, and AKT pathways in melanoma as a result of NMT-1 blockade [7]. In a uveal melanoma cells lines 92.1 and Mel290, we did not observe suppression of NMT-1 expression when treated for 24 hours with 2.7 μM Tris DBA. This suggests that the inhibitory effect might be independent of NMT-1 (Figure 3A). In fact, p-ERK was activated 24 to 48 hours after drug exposure and p-AKT activation was noted at 2 hours. P-FAK was not affected by the drug. We also examined via immunofluorescence expression of SRC and MARCKS, both involved in the myristoylation pathway, upon treatment with Tris DBA palladium at 5.5 μM for 24 hours. We observed no inhibition of either SRC or MARCKS. Remarkably, we saw increased signal of both proteins with treatment localized to the perinuclear area (Figure 3B). To examine whether Tris DBA palladium inhibits previously reported NMT-1 activity, uveal melanoma cell lines were treated with Tris DBA palladium at 5.5 μM and 10.9 μM for 24 hours and analyzed for NMT-1 activity (Figure 3C and 3D). We observed no significant NMT-1 inhibition in any of the cell lines tested. The xenograft tumors were analyzed for activity and no NMT-1 inhibition was present when mice were given Tris DBA palladium feed for a time period of 14 days.

### Tris DBA palladium inhibits ARF6 activity

**Figure 4:**
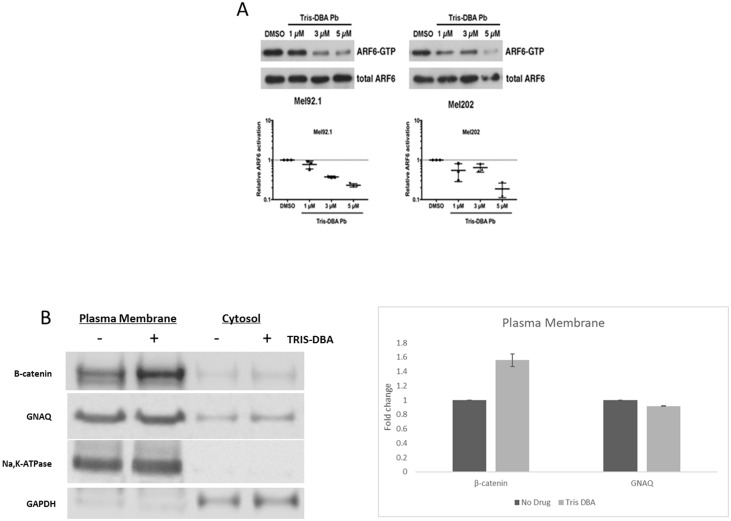
Tris DBA inhibits ARF6 activity and localizes B-catenin to plasma membrane. (**A**) ARF6-GTP pulldown assay was performed on *in vitro* Mel92.1 and Mel202 cell lines to collect protein indicative of AFR6 activity. Protein collected from pulldown assay was then used to perform Western Blot analysis and compared to DMSO control protein levels which revealed significant inhibition of ARF6 activity at 3 uM in the Mel92.1 cell line and 5 uM in the Mel202 cell line. (**B**) Plasma membrane and total membrane fractions isolated from 92.1cells treated with no drug or Tris DBA for 24 hours at 2 μM concentration. Cells were fractionated to collect plasma membrane. Western Blot of B-catenin and GNAQ is shown. Na,K-ATPase was used as loading control for plasma membrane fractions and GAPDH was used as the loading control for cytosol fractions. Briefly, 100 × 10^6^ cells treated with no drug or Tris DBA were collected and homogenized. Cells were centrifuged and the supernatant (cytosol) was collected. The remaining pellet was purified according to manufacturer instructions (Abcam) for plasma membrane fractions. Quantitation of B-catenin and GNAQ from plasma fractions was performed using Image Studio Lite from LI-COR Biosciences.

To determine the effect of Tris DBA palladium on ARF6 activation, we performed ARF6-GTP pulldown assays on Mel92.1 and Mel202 uveal melanoma cell lines that had been treated with concentrations of Tris DBA palladium ranging from 1 to 5 μM (Figure 4). For both cell lines, we observed a concentration-dependent reduction in ARF6 activation (ARG6-GTP) without a change in total ARF6 expression that reached statistical significance at 3 μM for Mel92.1 and 5 μM for Mel202. These results indicate that Tris DBA palladium can inhibit the activation of ARF6 in these uveal melanoma cell lines. It has been reported that GNAQ and ARF6 control the subcellular localization and transactivation of β-catenin in uveal melanoma cells [8]. Since ARF6 activity was inhibited by Tris DBA, we examined the subcellular localization of β-catenin expression and found increased β-catenin levels in the plasma membrane after treatment with 2 μM Tris DBA treatment for 24 hours (Figure 4B).

### Tris DBA palladium induces apoptosis in GNAQ uveal melanoma

**Figure 5:**
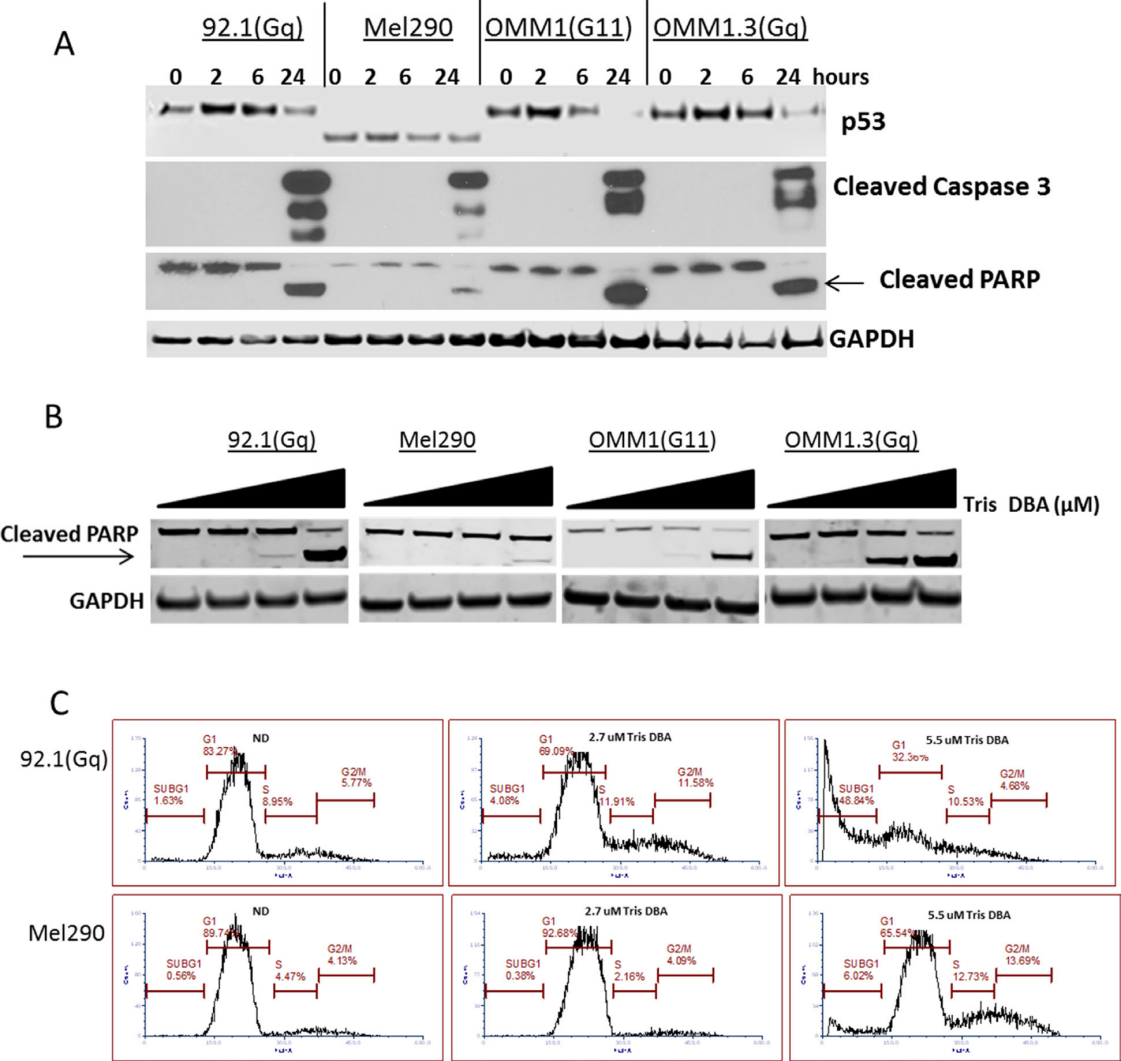
Tris DBA induces apoptosis in GNAQ mutant uveal melanoma. (**A**) Western Blot of p53, cleaved caspase 3, and cleaved PARP at 0, 2, 6, and 24 hours in uveal melanoma cells is shown using 5.5 μM Tris DBA concentration. GAPDH was used as a loading control. 92.1(Gq mutant), Mel290 (Wild Type), OMM1 (G11 mutant) and Omm1.3(Gq mutant) uveal melanoma cell lines were treated with Tris DBA and lysates were collected in RIPA buffer. Protein concentrations were determined and 30 μg of protein was loaded onto a gradient gel. Western Blot was then performed on proteins of interest. (**B**) Dose dependent western blot of cleaved PARP using increasing concentrations of 0, 1.1, 2.6 and 5.5 μM of Tris DBA for 24 hours. 92.1(Gq mutant), Mel290 (Wild Type), OMM1 (G11 mutant) and Omm1.3 (Gq mutant) uveal melanoma cell lines were treated with Tris DBA and lysates were collected in RIPA buffer. Protein concentrations were determined and Western Blot was then performed on proteins of interest. (**C**) Cell cycle analysis with propidium iodide DNA staining using 2.7 uM and 5.5 μM Tris DBA treated uveal melanoma cells after 72 hours of treatment. 92.1(Gq mutant) and Mel290 (Wild Type) were treated with Tris DBA. Cells were fixed in 70% ethanol and kept at –20° C. Cells were stained with propidium iodide and analyzed by flow cytometry on a BD Calibur. Analysis was performed using FCS Express 6.

We next investigated if growth inhibition correlated with apoptosis. We observed caspase3 activation and PARP cleavage after 24 hours of exposure with 5.5μM in all the cell lines tested (Figure 5A). This effect was concentration dependent with the largest sub-G1 peak noted with 5.5 μM of drug (Figure 5C) and also by PARP cleavage induction at similar concentration (Figure 5B). Interestingly, GNAQ WT cell line MEL290 showed less caspase activation and PARP cleavage by western blot. This was confirmed by flow cytometry with 5.5 μM of Tris DBA Palladium (Figure 5C).

### Gene array of vehicle and treated tumors

Genes reported upregulated by Tris DBA palladium *in vivo* include GSTM2, which has been associated with improved prognosis in solid tumors and often demonstrates promoter hypermethylation in advanced cancer [9, 10]. A second upregulated gene observed here is IFI30/GILT, which has been implicated in regulation of immunity and autoimmunity to melanocyte antigens [11]. One of the most downregulated genes is Cyr61, a pro-inflammatory gene associated with tumorigenesis and IL-8 production [12]. Finally, Cry61 is an established target of ARF6 in uveal melanoma [8]. The transcription factor c-jun is also downregulated, and c-jun is an established target of ARF6 signaling [8] (Table 1).

**Table 1:** Gene array analysis of *in vivo* 92.1 xenograft tissues reveals gene profile changes following treatment with Tris DBA enriched food pellets

Gene symbol	*p*-value(Tris_DBA vs. control)	Fold-change(Tris_DBA vs. control)	Fold-change(Tris_DBA vs. control) (description)
LINC00342	0.0278894	1.88409	Tris_DBA up vs Control
MIR4267	0.0109544	1.67557	Tris_DBA up vs Control
OR1F1	0.00796116	1.58286	Tris_DBA up vs Control
GSTM2	0.0133839	1.5688	Tris_DBA up vs Control
PRKXP1	0.0283998	1.56663	Tris_DBA up vs Control
DDX12P	0.028791	1.56372	Tris_DBA up vs Control
HERC2P4	0.0492184	1.5589	Tris_DBA up vs Control
SNORD70	0.0402876	1.53475	Tris_DBA up vs Control
LOC100286922	0.00159986	1.53324	Tris_DBA up vs Control
ITPKB-IT1	0.0239881	1.51584	Tris_DBA up vs Control
NPPA-AS1	0.00862237	1.50476	Tris_DBA up vs Control
IFI30	0.0167487	1.49128	Tris_DBA up vs Control
LOC729739	0.00231828	1.48708	Tris_DBA up vs Control
MNS1	0.0403036	1.47866	Tris_DBA up vs Control
HMGN5	0.0252726	1.47344	Tris_DBA up vs Control
LOC105369685	0.0385378	1.47258	Tris_DBA up vs Control
CDK5	0.0253059	1.46905	Tris_DBA up vs Control
NSA2	0.0322093	1.46794	Tris_DBA up vs Control
EME1	0.00379792	1.46696	Tris_DBA up vs Control
IGHV1OR21-1	0.00997831	1.45999	Tris_DBA up vs Control
MIR4782	0.0120561	1.44693	Tris_DBA up vs Control
GSTT2	0.0414451	1.44095	Tris_DBA up vs Control
ADRB2	0.0464329	1.43553	Tris_DBA up vs Control
TPTE2P1	0.0122935	1.42744	Tris_DBA up vs Control
ARL17A	0.01489	1.42696	Tris_DBA up vs Control
RXRA	2.70E-05	1.42626	Tris_DBA up vs Control
HAGLR	0.00426283	1.41826	Tris_DBA up vs Control
KLHL38	0.00872755	1.41106	Tris_DBA up vs Control
ZNF700	0.00153304	1.40695	Tris_DBA up vs Control
MACROD1	0.0361136	1.4061	Tris_DBA up vs Control
CHST12	0.0421867	1.403	Tris_DBA up vs Control
GUSBP1	0.0338928	1.40203	Tris_DBA up vs Control
ARSA	0.0241483	1.40066	Tris_DBA up vs Control
SNORA11	0.0228449	−3.92074	Tris_DBA down vs Control
JUN	0.0276787	−3.03041	Tris_DBA down vs Control
SCARNA4	0.0161854	−2.15055	Tris_DBA down vs Control
CYR61	0.0116602	−1.96086	Tris_DBA down vs Control
SNORD115-22	0.0371072	−1.90193	Tris_DBA down vs Control
RGS2	0.0455401	−1.86268	Tris_DBA down vs Control
SCARNA2	0.00236968	−1.80903	Tris_DBA down vs Control
SNORD115-11	0.0465115	−1.76255	Tris_DBA down vs Control
SNORD115-11	0.0465115	−1.76255	Tris_DBA down vs Control
SNORD115-11	0.0465115	−1.76255	Tris_DBA down vs Control
SNORD115-11	0.0465115	−1.76255	Tris_DBA down vs Control
SNORD115-9	0.0465115	−1.76255	Tris_DBA down vs Control
SNORD115-9	0.0465115	−1.76255	Tris_DBA down vs Control
SNORD115-5	0.0465115	−1.76255	Tris_DBA down vs Control
TAS2R46	0.0391203	−1.75125	Tris_DBA down vs Control
MIR1305	0.00837122	−1.66914	Tris_DBA down vs Control
VIM-AS1	0.0096019	−1.64823	Tris_DBA down vs Control
MIR374A	0.014889	−1.63399	Tris_DBA down vs Control
SNORD115-39	0.0369375	−1.63077	Tris_DBA down vs Control
HSPA6	0.0352236	−1.62132	Tris_DBA down vs Control
SNORA71D	0.0413299	−1.61351	Tris_DBA down vs Control
LINC00312	0.0019944	−1.6131	Tris_DBA down vs Control
EGR1	0.0102042	−1.60692	Tris_DBA down vs Control
MIR3175	0.0153444	−1.54887	Tris_DBA down vs Control
SNORD115-16	0.0283855	−1.47207	Tris_DBA down vs Control
SNORD115-1	0.0283855	−1.47207	Tris_DBA down vs Control
SCARNA18	0.0401323	−1.46856	Tris_DBA down vs Control
HMOX1	0.0108223	−1.45584	Tris_DBA down vs Control
PGK1	0.0441437	−1.45513	Tris_DBA down vs Control
HSF2BP	0.0213714	−1.45052	Tris_DBA down vs Control
HIST2H3D	0.0490749	−1.43962	Tris_DBA down vs Control
TNFRSF12A	0.0043647	−1.43926	Tris_DBA down vs Control
JUNB	0.00515271	−1.43517	Tris_DBA down vs Control
SNORD104	0.0225254	−1.43481	Tris_DBA down vs Control
11-Sep	0.00318014	−1.43245	Tris_DBA down vs Control
MAFF	0.00447969	−1.42909	Tris_DBA down vs Control
SCARNA8	0.0188127	−1.4263	Tris_DBA down vs Control
HSP90AA6P	0.0236067	−1.4202	Tris_DBA down vs Control
PTPRZ1	0.0243325	−1.41038	Tris_DBA down vs Control
LOC100129291	0.0364835	−1.40793	Tris_DBA down vs Control
LINC00910	0.000541337	−1.40159	Tris_DBA down vs Control

Gene array analysis was performed on 92. 1 The triplicate biological samples of tumors from both Tris DBA PD fed mice and Control fed mice were analyzed by NOVA statistical testing for significance *p* values < 0.05. Data are normalized to the control treated tumors and presented as fold-change.

## DISCUSSION

Uveal melanoma is the leading cause of death due to a condition of the eye [13–15]. Like cutaneous melanoma, uveal melanoma arises from melanocytes, but differs in many substantial ways from cutaneous melanoma. First, uveal melanoma is often detected late because of its anatomic location. Second, it has a very strong propensity for liver metastases, as opposed to lymph node, lung, and brain in cutaneous melanoma [14, 15]. Third, the majority of uveal melanoma is driven by mutations in GNAQ and GNA11, as opposed to Braf and Nras mutations in cutaneous melanoma [16, 17]. Fourth, uveal melanoma lacks a common UV signature and contains a far smaller mutational burden compared with cutaneous melanoma. These features have made the advances that have benefitted patients with cutaneous melanoma, such as Braf inhibition and immunotherapy less relevant for the patient with uveal melanoma.

Current treatments include surgery, dacarbazine, and ipilimumab, but are not highly efficacious [13]. Experimental approaches such as selumetinib, an orally active MEK1/2 inhibitor, has resulted in increases in progression free survival, but not overall survival [18]. Combination trials of MEK inhibitors with protein kinase C inhibitors are ongoing [19]. Carbozatinib, a small molecule inhibitor of MET and VEGF, has been recently evaluated and is now being tested in combination with temozolomide [20]. Failure of therapies is likely linked to plasticity of signaling, with rapid adaptation of the tumor to alternate signaling pathways upon exposure to signal transduction inhibitors [21].

Advances in the biology of uveal melanoma have been made in recent years. These include discovery of the major driver mutations in uveal melanoma, and prognostic factors for metastatic spread, including monosomy 3 and BAP1 mutation [22–24]. A prognostic panel has subdivided uveal melanoma into class 1, which has a high level of melanocytic differentiation and better prognosis, compared with class 2, which is more poorly differentiated and prone to metastatic spread.

Recently ARF6 has been found to be a major signaling node in GNAQ mutant melanoma, and blockade of ARF6 has been shown to inhibit multiple pathways activated in uveal melanoma [8]. ARF6 potentiates hepatocyte growth factor/MET signaling, which is a key player in the high rate of hepatic metastasis seen in uveal melanoma [25, 26]. ARF6 is also associated with potentiation of additional growth factor receptors, including epidermal growth factor receptor (EGFR) signaling, and has been associated with beta 1 integrin signaling and pancreatic cancer metastasis to the liver [27]. Liver metastasis is a common site for solid tumor metastasis, including lung, breast, pancreatic, and uveal melanoma, and ARF6 activation might be a common thread, as ARF6 activation has been shown to be an adverse prognostic factor in these common malignancies [28, 29]. Of interest, Tris DBA was able to significantly prevent liver metastases of pancreatic cancer [7], and similar signaling processes might underlie hepatic metastases from uveal melanoma.

Tris DBA palladium is a small molecule organometallic compound that has been shown to have activity against multiple preclinical models, including cutaneous melanoma, pancreatic carcinoma, multiple myeloma, and chronic lymphocytic leukemia [6, 7], [30]. Among its previously described modes of action is inhibition of N-myristoyltransferase 1, an enzyme that catalyzes the addition of myristoyl side chains to substrates such as src family kinases, for which cellular localization is required for optimal activity [31, 32]. Tris DBA palladium also inhibited the orthotopic growth and liver metastatic growth of pancreatic carcinoma. In this study, we demonstrate the efficacy of Tris DBA palladium *in vitro* and *in vivo* against uveal melanoma. Most importantly, we demonstrate that Tris DBA palladium inhibits ARF6 activation. Finally, we demonstrate that Tris DBA palladium is orally active against GNAQ mutant uveal melanoma *in vivo*.

Tris DBA Palladium decreased proliferation in all cell lines, but most drastically in the GNAQ mutant cell lines. However, at an effective dose of 2.7 μM, we did not see reductions in phosphorylated ERK, Akt, FAK, or total NMT1 levels, implying that Tris DBA was acting independently of these pathways. Tris DBA Palladium caused membrane localization of beta catenin and downregulated Cyr61, both of which are observed upon ARF6 inhibition [8]. However, Tris DBA palladium likely acts through additional ARF6-independent pathways because other factors that have been shown to be downstream of oncogenic GNAQ-ARF6 signaling, such as ERK1/2 [8], are regulated differently in response to Tris DBA palladium. Tris DBA palladium downregulates Cyr61 *in vivo*, which is associated with inflammatory tumorigenesis, including resistance to chemotherapy and IL-8 production. In addition, a large cohort of small nucleolar RNAs is downregulated. The precise significance of this is not known, but SNORA11 is involved in conversion of pseudouridine to uridine [33]. Several other small nucleolar RNAs are also downregulated, including the SNORD115 family. This suggests that oral Tris DBA palladium appears to have a major impact in tumor RNA splicing. Finally junB was downregulated by Tris DBA palladium, and junB has been shown to mediate pro-tumorigenic effects of TGF beta [34].

Tris DBA is a new and potent anticancer agent. Its intravenous development has been hampered by its poor solubility. In this manuscript, we demonstrate that Tris DBA is orally bioavailable and active against a GNAQ mutant preclinical model of uveal melanoma. While there are multiple targeted therapies for Braf mutant melanoma, development of novel therapies for uveal melanoma lag far behind. In addition to its oral bioavailability, we demonstrate that its mechanism by oral delivery is distinct from its previous mechanism of action against melanoma when given intraperitoneally [35]. This raises the possibility that one could potentially avoid drug resistance to a particular compound by changing its mode of delivery [36]. IND enabling studies and clinical development of Tris DBA are warranted.

## MATERIALS AND METHODS

### Cell culture

All cell lines were maintained in RPMI1640 supplemented with heat inactivated 10% Fetal Bovine serum, 100 units/ml penicillin and 100 ug/ml streptomycin and maintained at 37° C and 5% CO2. 92.1 cells were provided by Dr. William Harbour (Washington University, St. Louis, MO). Omm1.3 was provided by Dr. Bruce Ksander (Harvard Medical School, Boston, MA). Omm1 was kindly provided by Dr. Boris Bastian (University California of San Francisco, San Francisco, CA). Mel290 was from David Folberg (University of Illinois, Chicago, IL).

### Cell viability assays

Cells were plated in a 96-well plate and treated with Tris DBA or DMSO at indicated concentrations for a period of 24 hrs. Viability was assessed using Cell Counting Kit from Dojindo Molecular Technologies (Rockville, MD, USA) as per manufacturer’s instructions. Briefly, CCK-8 reagent was added after drug treatment to the cells and allowed to incubate at 37º C for 1 hour. The OD was read at 450 nm and drug treated samples were presented as percent of control.

### Western blots

Cells were lysed in radioimmunoprecipitation assay (RIPA) buffer supplemented with protease inhibitor cocktail tablet (Roche Diagnostics) and 1 mmol/L Na3VO4. Equal amounts of protein were loaded and separated on a 4–12% PAGE gel (Invitrogen). Proteins were transferred to polyvinylidenedifluoride (PVDF) membranes, which were blocked in 5% nonfat dried milk. Membranes were then incubated with primary and secondary antibody and developed by ECL. Antibodies used to probe were NMT1 and GNAQ (Santa Cruz Biotechnology), phospho-p44/42 MAPK (ERK1/2), Total p44/42 MAPK (ERK1/2), phospho-Akt (Ser473), phospho-FAK (Tyr576/577), Na,K-ATPase, GAPDH, Caspase-3, and Cleaved PARP (Cell Signaling).

### Flow cytometry and cell cycle

Cell cycle analysis was performed after 72 hours of treatment. Cells were fixed in 70% ethanol and kept at −20º C for overnight or longer. Next day, cells were stained with propidium iodide and MPM-2 antibody for mitotic population distinction and analyzed by flow cytometry on a BD Calibur. Analysis was performed using FCS Express 6.

### Immunofluorescence

Cells were treated with Tris DBA for 24 hours and rinsed three times in PBS, fixed with acetone: methanol (1:1) at −20º C for 10 minutes and blocked in 3% BSA for 1 hour at room temperature. Cells were incubated with primary antibodies overnight at 4º C. Next day, cells were incubated in fluorescently conjugated secondary antibody for 1 hour at room temperature. Cells were mounted in Prolong Gold antifade reagent with DAPI for nuclear staining. Images were captured using a Nikon T1 Eclipse inverted microscope with a CFI Apochromat TIRF 60XC Oil lens. Images were analyzed with Image J.

### Plasma membrane protein extraction

Plasma membrane and total membrane fraction was isolated with Plasma Membrane Protein Extraction Kit (Abcam). Briefly, 100 × 10^6^ cells were treated with no drug or Tris DBA for 24 hours. Cells were scraped in PBS and collected, and homogenization was completed with a Dounce homogenizer. Cells were centrifuged and the supernatant (cytosol) was collected. The remaining pellet was purified according to manufacture instructions and plasma membrane proteins were collected. B-catenin and GNAQ quantitation of plasma fractions was performed using Image Studio Lite from LI-COR Biosciences

### N-myristoyltransferase activity assay

N-myristoyltransferase activity was assayed as described earlier [30, 31]. Briefly, 20 μg of total protein lysate was used and the myristolation reaction was initiated by the addition of freshly generated [3H]myristoyl-CoA. The samples were incubated 30° C for 30 min. The reaction was terminated and radioactive incorporation was determined by the liquid scintillation counting. The background was subtracted from the assays (performed in triplicate) and the NMT activity (mean ± S.D) was expressed as 1 fmol of myristoyl peptide formed per mg protein per min.

### ARF6-GTP pulldown assay

ARF6-GTP pulldown assays were performed according to manufacturer’s instructions using the Arf6 Activation Assay Kit (Cell Biolabs). Briefly, Mel92.1 or Mel202 cells were grown in RPMI-1640/10% FBS to 80% confluency in 10 cm tissue culture dishes and then treated with 1 μM to 5 μM Tris DBA palladium or vehicle (0.1% DMSO in medium) for 3 h at 37° C/5% CO_2_. Cells were lysed at 4° C in 1X lysis buffer (Cell Biolabs kit) supplemented with protease and phosphatase inhibitor cocktail (ThermoFisher Scientific). Total cell lysate (0.5 mg) was incubated with GGA3-conjugated agarose beads for 1 hour, and then the beads were washed three times with 1X lysis buffer supplemented with protease and phosphatase inhibitors. Precipitates with beads (ARF6-GTP) and total cell lysates (total ARF6) were analyzed by western blot using 5% nonfat dry milk in PBST as a blocking agent, anti-ARF6 as a primary antibody (diluted 1/1000; Cell Signaling Technology), and a secondary antibody conjugated to horseradish peroxidase (diluted 1/5000; Jackson ImmunoResearch). Signals were detected using Immobilon Western Chemiluminescent HRP Substrate (Millipore). Quantification was performed using scanning densitometry and ImageJ (NIH), and ARF6-GTP levels were normalized to total ARF6 levels.

### Xenograft studies

6–8 week nu/nu SCID female mice bearing subcutaneously injected 92.1 tumors (7 mice/group) of 100 mm3 diameter were treated with vehicle Tris DBA feed (660 ppm (https://www.testdiet.com/). After 2 weeks, two animals from each group were sacrificed and tumors were collected for analysis. Tumors were measured every 2 to 3 days with calipers, and tumor volumes were calculated by the formula 4/3 × r3 [r = (larger diameter + smaller diameter)/4. Toxicity was monitored by weight loss. Experiments were carried out under institutional guidelines addressing the proper and humane use of animals. The Memorial Sloan- Kettering Cancer Center Animal Care and Use Committee and Research Animal Resource center approved this study. The study is also in accordance of the Principles of Laboratory Animal Care (NIH Publication No. 85-23, released 1985).

### Gene array and whole-transcriptome expression analysis

Gene Array of vehicle and drug treated tumors was performed as previously described [36]. Briefly, RNA was extracted using the Qiagen miRNEasy kit w/ on column DNAse treatment as described by the manufacturer. Tissue was lysed and homogenized in Qiazol buffer homogenizer for 40 seconds or until fully disrupted (1 ml Qiazol per 100 mg of tissue). RNA was eluted in 50 ul nuclease free water. 1 ul was used to determine OD (optical density) values on a Nanodrop 1000. 1ul was used to assess sample profiles on the Agilent 2100 using the RNA 6000 Nano assay. 250 ng of total RNA was amplified and labeled using the ThermoFisher Scientific Illuina™ TotalPrep™ RNA Amplification kit according to the manufacturer’s protocol. Labeled cRNA was hybridized to Illumina HT12 bead array according to the protocol described in the WGGEX Direct Hybridization Assay user guide. Image acquisition and data extraction were performed with an Illumina HiScan laser scanner and GenomeStudio software as previously described [36].

### Statistics

Comparisons were performed using an unpaired, two-tailed, unequal variance Student’s *t*-test. Probability values less than 0.05 were considered significant. Data are presented as mean ± SEM. For ARF6 pulldown assays, a one-way randomized block ANOVA with Dunnett’s multiple comparison test was performed following log transformation of the data. Illumina HiScan laser scanner and GenomeStudio software.

## SUPPLEMENTARY MATERIALS


