# Supplementary material for: Tris DBA palladium is an orally available inhibitor of GNAQ mutant uveal melanoma in vivo
Source: Oncotarget. 2019 Jul 9;10(43):4424–36. doi: 10.18632/oncotarget.27040 (PMC6633893; doi:10.18632/oncotarget.27040)
Supplement: Supplementary file 1 [file oncotarget-10-4424-s001.pdf]

Tris DBA palladium is an orally available inhibitor of GNAQ mutant uveal melanoma *in vivo*

SUPPLEMENTARY MATERIALS

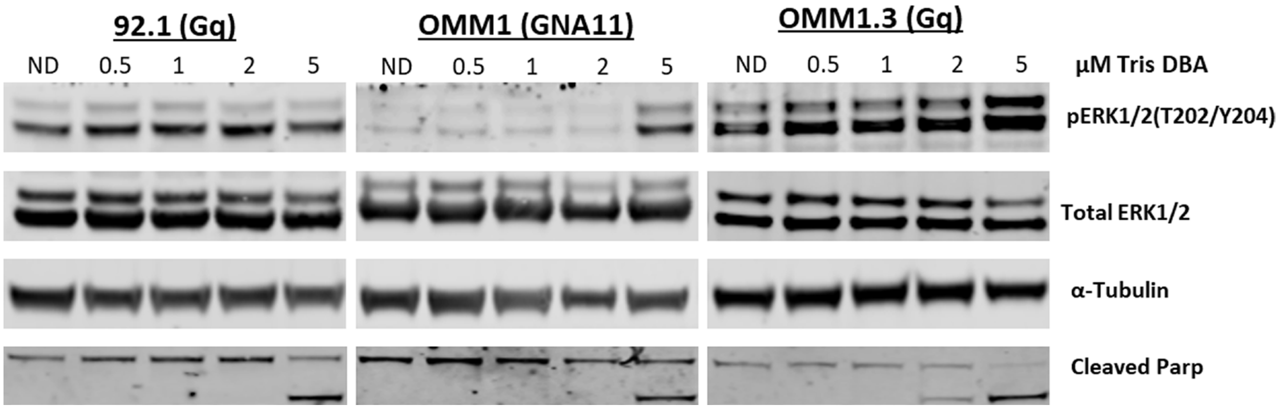

Supplementary Figure 1: Tris DBA inhibits cell growth independent of MAPK pathway in GNAQ/GNA11 uveal melanoma cells. Western Blot of phospoho-ERK1/2 (Thr202/Tyr204), Total ERK1/2, α- Tubulin and cleaved PARP at 24 hours is shown at 0, 0.5, 1, 2, and 5 µM Tris DBA. Briefly, 92.1(Gq mutant). OMM1 (GNA11 mutant) and Omm1.3 (Gq mutant) uveal melanoma cell lines were treated with Tris DBA and lysates were collected in RIPA buffer. Protein concentrations were determined and 30ug of protein was loaded onto a gradient gel. Western Blot was then performed on proteins of interest.
